# Paroxysmal Amnesia Attacks due to Hashimoto's Encephalopathy

**DOI:** 10.1155/2016/1267192

**Published:** 2016-02-29

**Authors:** Pelin Nar Senol, Aylin Bican Demir, Ibrahim Bora, Mustafa Bakar

**Affiliations:** Department of Neurology, Faculty of Medicine, Uludag University, 16285 Bursa, Turkey

## Abstract

Hashimoto's encephalopathy is a rare disease which is thought to be autoimmune and steroid responsive. The syndrome is characterized by cognitive impairment, encephalopathy, psychiatric symptoms, and seizures associated with increased level of anti-thyroid antibodies. The exact pathophysiology underlying cerebral involvement is still lesser known. Although symptoms suggest a nonlesional encephalopathy in most of the cases, sometimes the clinical appearance can be subtle and may not respond to immunosuppressants or immunomodulatory agents. Here we report a case who presented with drowsiness and amnestic complaints associated with paroxysmal electroencephalography (EEG) abnormalities which could be treated only with an antiepileptic drug.

## 1. Introduction

Hashimoto's encephalopathy (HE) has been acknowledged as a syndrome of altered mental status varying between delusional thinking, confusion, and hallucinations with the presence of increased serum levels of anti-thyroid antibodies. Epileptic seizures may accompany these symptoms. The syndrome was first described by Brain and colleagues as a case report in 1966 [1]. HE syndrome can be acute and fulminant or chronic as in dementia [2]. Ataxia, tremor, myoclonus, focal neurologic deficit, and some psychiatric symptoms including depression, manic state, and psychosis in severe forms can be observed in this syndrome [3, 4]. HE is relatively rare and still lesser known. Here we present a patient with paroxysmal memory impairment attacks and loss of attention. Here we report and discuss clinical, electroencephalographic (EEG), and neuroimaging findings of a HE syndrome by highlighting diagnostic and treatment features.

## 2. Case Presentation

**Figure 1:**
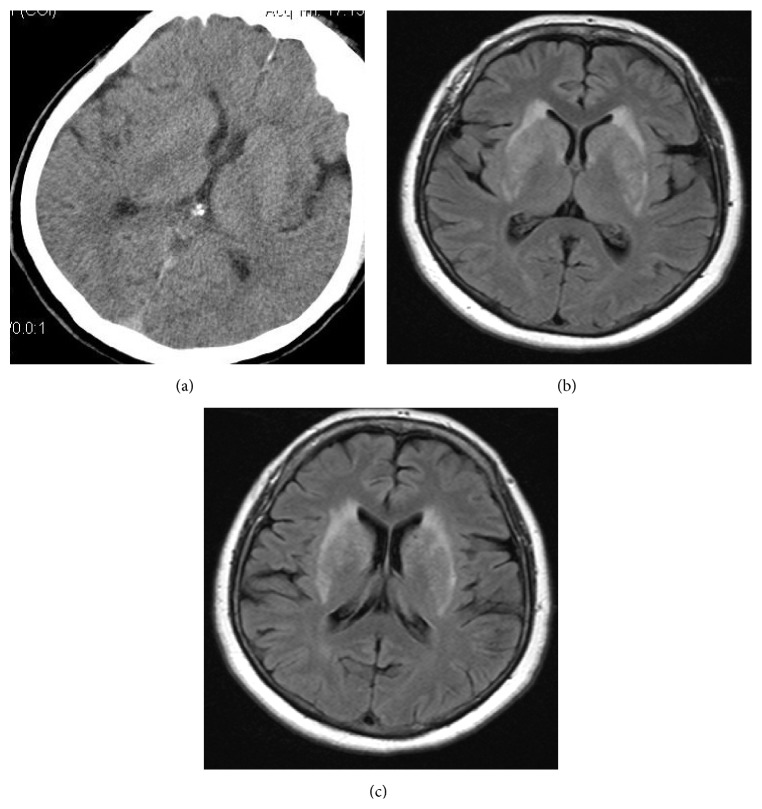
Noncontrast CT scan is normal (a) and FLAIR (fluid-attenuated inversion recovery) images demonstrate increased T2 signal activity at bilateral lentiform and caudate nucleus (b-c).

**Figure 2:**
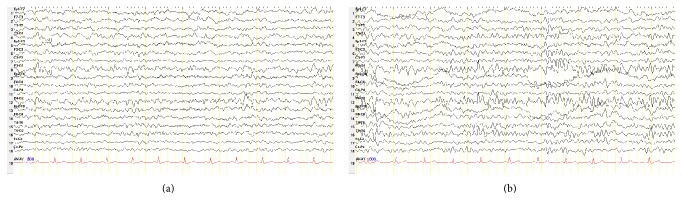
EEG showing the presence of slow and irregular background (a) and bilateral sharp wave paroxysms (b).

A 52-year-old woman suffering with drowsiness and memory problems was admitted to our hospital's neurology department. She complained about disremembering some parts of a day and what she had done. These attacks were lasting for a few minutes to one hour sometimes and repeating every day in last three months. She had no history of psychiatric disease, epilepsy, or other mental problems including dementia. Neurologic examination did not reveal any focal abnormalities. Extended laboratory findings including CBC, blood urea nitrogen, creatinine, liver function tests, C-reactive protein, erythrocyte sedimentation rate, vitamin B12, folate, electrolytes, blood ammonia, and glucose levels were normal. Thyroid function tests including free T3, free T4, and TSH were normal. Her mini mental state examination test score was within the normal range for her age and education level (29/30). Neither a noncontrast cranial computerized tomography (Figure 1(a)) nor the T1-weighted images of cranial magnetic resonance imaging (MRI) revealed abnormalities like calcification. T1-weighted images after intravenous gadolinium administration were unremarkable. No evidence of cytotoxic edema was present on diffusion-weighted images (DWI) and apparent diffusion coefficient (ADC). Fluid-attenuated inversion recovery (FLAIR) and T2 weighted images demonstrated bilaterally symmetric basal ganglia hyperintensity (Figures 1(b) and 1(c)). On EEG, the background activity was mildly slow and irregular (Figure 2(a)). An interictal EEG recording showed bilateral paroxysms of rudimentary spike and sharp wave discharges (Figure 2(b)). Mini mental state examination test score was 27. To explain the etiology of subacute encephalopathy we examined her autoimmune markers. Serum anti-nuclear antibodies, anti-dsDNA, anti-neutrophil cytoplasmic antibody, and ENA panel tests were negative. Anti-thyroid antibodies were elevated. Anti-TPO was 1258 U/mL and the anti-TG antibody was 154. In the light of clinic and laboratory findings she was diagnosed as HE. Intravenous methylprednisolone treatment with a dose of 1000 mg per day was initiated and maintained orally 60 mg per day. Control EEG on the 14th day of the treatment showed a normal background activity. Her attention and drowsiness were also improved. However the paroxysms of sharp waves on EEG persisted and she was still complaining about amnesia attacks. The patient was given orally levetiracetam with a dose of 1000 mg per day. On follow-up she was symptom free and her control EEG recording was normal.

## 3. Discussion

Hashimoto's thyroiditis can cause an autoimmune encephalopathy with an unknown pathophysiology [5]. The clinic presentation of this rarely seen syndrome is variable including psychiatric symptoms, cognitive impairments, seizures, and hemispheric neurologic deficits [6]. Patients with HE can be euthyroid as in our case [7]. An extended diagnostic procedure for vascular, toxic, metabolic, and infectious diseases should be performed carefully to avoid other etiologies which are not caused by thyroid dysfunctions such as autoimmune encephalopathy, infections of central nervous system, acute disseminated encephalomyelitis (ADEM), Creutzfeldt-Jakob disease, Wernicke's encephalopathy, and CNS angiitis [6].

The most common cranial MRI changes in this syndrome include bilateral subcortical high signal lesions and bilateral hippocampus or thalamic lesions [7]. However, as in a recently reported case [8] our patients MRI showed bilaterally, symmetrical basal ganglia hyperintensity without any mass effect, cytotoxic edema, and calcification.

Our case is a patient who presented with drowsiness, loss of attention, and paroxysmal memory impairment attacks associated with symmetric slowing background activity and bilateral paroxysms of sharp wave discharges on EEG. It is well known that HE may be associated with mental and cognitive dysfunctions [9]. In a recent study, Wang et al. showed that the impairments in cognitive functions for patients with HE are similar to those with Alzheimer's disease patients [10]. Epileptic seizures can also be seen in HE. The most frequent seizure types contain generalized tonic-clonic seizures (GTCS) and complex partial seizures often associated with an EEG showing diffuse slowing background activity [5]. The syndrome can cause status epilepticus rarely [11, 12]. In HE, steroid therapy has shown to be associated with improvement or resolution of EEG findings. However, in our patient even though the slow background activity on EEG was improved after steroid therapy, the paroxysmal slow-wave discharges persisted until levetiracetam was initiated as an antiepileptic drug. Similar to our case, in two cases recently reported by Wang et al, levetiracetam was found to be effective in treatment of HE [10].

It is important to recognize this syndrome which is usually treatable successfully with corticosteroids and other immunosuppressive agents such as IVIG, methotrexate, mycophenolate, and azathioprine [13]. Nevertheless, as in our case steroid therapy may not always be able to treat both clinic and electrophysiologic symptoms. Therefore appropriate antiepileptic medication should be administered.

HE should be considered especially in patients with encephalopathy with an unexplained etiology. But our case demonstrated that HE symptoms can be subtle. On such an occasion, the clinicians should suspect the presence of HE and evaluate anti-thyroid antibodies. But we thought that emphasis must be given to EEG which is valuable in evaluation of neurological conditions either associated with or apart from epilepsy [14]. As in our case, in patients with impaired consciousness or altered mental state EEG is important in diagnosing and monitoring the whole treatment process.
